# Natural Dietary Compound Xanthohumol Regulates the Gut Microbiota and Its Metabolic Profile in a Mouse Model of Alzheimer’s Disease

**DOI:** 10.3390/molecules27041281

**Published:** 2022-02-14

**Authors:** Wei Liu, Kaiwu He, Desheng Wu, Li Zhou, Guowei Li, Zequn Lin, Xifei Yang, Jianjun Liu, Maggie Pui Man Hoi

**Affiliations:** 1 State Key Laboratory of Quality Research in Chinese Medicine, Institute of Chinese Medical Sciences, University of Macau, Macau 999078, China; liuweiszcdc@163.com; 2 Shenzhen Key Laboratory of Modern Toxicology, Shenzhen Medical Key Discipline of Health Toxicology (2020–2024), Shenzhen Center for Disease Control and Prevention, Shenzhen 518055, China; kaiwuhe@gmail.com (K.H.); dswucn@126.com (D.W.); zhouliszcdc@126.com (L.Z.); l18200702395@163.com (G.L.); manta6@163.com (Z.L.); xifeiyang@gmail.com (X.Y.)

**Keywords:** xanthohumol, gut microbiota, Alzheimer’s disease, 16S rDNA sequencing, microbial diversity

## Abstract

Discovering new and effective drugs for the treatment of Alzheimer’s disease (AD) is a major clinical challenge. This study focuses on chemical modulation of the gut microbiome in an established murine AD model. We used the 16S rDNA sequencing technique to investigate the effect of xanthohumol (Xn) on the diversity of intestinal microflora in 2-month- and 6-month-old APP/PS1 mice, respectively. APP/PS1 and wild-type mice were treated by gavage with corn oil with or without Xn every other day for 90 days. Prior to and following treatment, animals were tested for spatial learning, cognitive and memory function. We found Xn reduced cognitive dysfunction in APP/PS1 mice and significantly regulated the composition and abundance of gut microbiota both in prevention experiments (with younger mice) and therapeutic experiments (with older mice). Differential microflora *Gammaproteobacteria* were significantly enriched in APP/PS1 mice treated with Xn. *Nodosilineaceae* and *Rikenellaceae* may be the specific microflora modulated by Xn. The penicillin and cephalosporin biosynthesis pathway and the atrazine degradation pathway may be the principal modulation pathways. Taken together, oral treatment with Xn may have a neuroprotective role by regulating the composition of intestinal microflora, a result that contributes to the scientific basis for a novel prophylactic and therapeutic approach to AD.

## 1. Introduction

Alzheimer’s disease (AD) is a progressive neurodegenerative disease that lays a heavy burden on society [1,2]. AD is characterized by continuous cognitive decline and worsening of daily living performance. With increased global population aging, it is estimated that AD will affect 82 million by 2030 and 152 million by 2050 if no effective therapeutic strategies are found [3]. Despite three decades of research, no drug has been shown to inhibit the development of AD. As a result, more and more attention is being paid to the discovery of drugs and other treatments for AD in the hope of effectively improving quality of life and reducing morbidity and mortality.

In recent years, the relationship between intestinal microecology and neurological disorders has attracted research attention [4,5]. Since the composition of intestinal microflora has been related to the pathogenesis of AD, regulation of gut microbiota may offer a new route for the prevention and treatment of this neurodegenerative disease [6]. Changes in the composition of intestinal microflora may be related to the deposition of Aβ in the AD brain [7]. In a male mouse model of AD, gut microbiome diversity has been shown to modulate the innate immunity of the host and affect Aβ denaturation, such that the number of amyloid plaques was reduced when mice were raised in a sterile environment or administered a mixture of antibiotics [8]. Compared with healthy volunteers and patients with mild cognitive impairment (MCI), the intestinal flora of AD patients was different and the diversity decreased. In AD and MCI patients, thick-walled bacteria were decreased, proteobacteria were enriched, and the pathway of polysaccharide biosynthesis and metabolism was increased, while immune system-related pathways were reduced [9]. Thus, while AD is associated with intestinal micro-ecological imbalance, a causal relationship has yet to be established.

Natural products have been a major resource for new drugs because of their highly diversified chemical structures and often specific biological activities. The female inflorescences of *Humulus lupulus* L., commonly called hops [10], have long been used in Chinese traditional medicine to treat many disorders, such as sleep disturbances and lack of appetite. In recent years, xanthohumol (Xn), a prenylated flavonoid from hops, has received much attention for its antibacterial [11], neuroprotective [12,13], anti-cancer [14,15], anti-inflammatory [16] and anti-oxidant [17] properties. Treatment with Xn derivatives, α,β-dihydro-XN (DXN) and tetrahydro-XN (TXN), improved the composition of the gut microbiota and bile acid metabolism associated with obesity and metabolic syndrome (MetS) in a diet-induced animal model [18]. In a previous study [19], we found Xn significantly suppresses Aβ production and tau hyperphosphorylation in neuro N2a/APP cells via regulation of amyloid precursor protein (APP) processing and the glycogen synthase kinase-3β (GSK-3β) pathway. Here, we investigate the potentially protective effects of Xn on cognitive impairment in APP and presenilin 1 (PS1) double-transgenic mice, while screening key components of the gut microbiome via 16S rDNA sequencing.

## 2. Results

### 2.1. Xn Improves Cognitive Function in APP/PS1 Mice

To investigate whether Xn preventively and/or therapeutically relieves cognitive deficits, we tested 2-month- and 6-month-old transgenic APP/PS1 mice and their wild-type littermates with Xn or corn oil by oral gavage every other day for 90 days. Animals displayed no obvious adverse effects or body-weight loss during the period of Xn treatment (data not shown).

The novel object recognition (NOR) test was used to assess short-term recognition and memory functions of mice at baseline (prior to Xn treatment). The higher the recognition coefficient, the better the memory. There was a significant difference in the baseline of the therapy experiment, while no difference was found in the baseline of the prevention experiment (Supplementary Figure S1).

**Figure 1:**
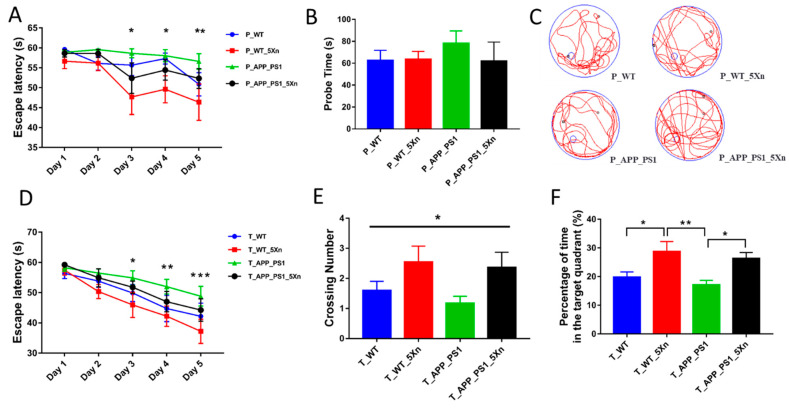
Xn improves behavioral performances of APP/PS1 mice. (**A**–**C**) Results of MWM test in the prevention experiment (*n* = 7–11 per group). (**D**–**F**) Results of MWM test in the therapy experiment (*n* = 10–14 per group). (**A**) Escape latency during platform trial test in the prevention experiment. As the learning time increased, the Xn-treated mice showed better memory compared to wild-type (WT) or APP/PS1 mice (* *p* < 0.05, ** *p* < 0.01, analyzed by repeated measures ANAVA). (**B**) Probe time of four groups in probe trial test. There was no difference in probe time among groups (*F* = 0.788, *p* = 0.538). (**C**) Representative tracing graphs of four groups in probe trial test. (**D**) Escape latency during platform trial test in the therapy experiment. As the learning time increased, the Xn-treated mice showed better memory compared to WT or APP/PS1 mice (* *p* < 0.05, ** *p* < 0.01, *** *p* < 0.001, analyzed by repeated measures ANAVA). (**E**) Crossing number of four groups in probe trial test. Significant difference was found in crossing number among groups (* *p* < 0.05, analyzed by two-way ANOVA). (**F**) Quantitative analysis of time spent in the target quadrant. Compared with WT or APP/PS1 mice, the Xn-treated mice showed better spatial recognition (* *p* < 0.05, ** *p* < 0.01, analyzed by two-way ANOVA). Error bar, SEM.

The Morris water maze (MWM) test was performed at the end of Xn treatment to evaluate learning and memory ability. Compared with untreated mice, Xn-treated animals in the prevention and therapy experiments performed better (Figure 1). During the 5-day training period, all the mice took less and less time to find the hidden platform in the prevention and therapy experiments (Figure 1A,D). Compared with wild-type (WT) mice, the APP/PS1 mice spent more time locating the underwater platform. However, Xn-treated mice spent less time than the Xn-untreated mice. These data indicate that Xn treatment enhanced learning ability. In the probe period, no obvious group differences were found in the swimming speed and total distance among groups, indicating that motor function was not impaired (data not shown). Compared with the WT group, APP/PS1 mice spent more time to probe the platform. Xn preventive treatment reduced the probe time (Figure 1B). Xn-treated mice crossed more frequently within the platform area and spent more in the target quadrant (Figure 1C,E,F). These results indicate that Xn treatment ameliorated cognitive decline in APP/PS1 mice.

### 2.2. Xn Regulates the Composition of the Intestinal Microbiome

To understand the changes of intestinal microflora before and after Xn administration, fecal samples from each group were analyzed by the 16S rDNA sequencing technique. After strict quality and size filtering, a total of 24546 Operational Taxonomy Units (OTUs) in 31 samples and 35507 OTUs in 48 samples were detected in the prevention and therapeutic experiments, respectively. We further found that 897 OTUs and 1041 OTUs co-existed among groups in the prevention and therapeutic experiments, respectively. We then analyzed the species taxonomy on the Family level and Genus level. Nineteen families, including *Muribaculaceae*, *Lactobacillaceae*, *Lachnospiraceae*, *Ruminococcaceae*, *Bacteroidaceae*, *Prevotellaceae* and *Erysipelotrichaceae*, formed the dominant microflora (Supplementary Figure S2A,B). Seventeen genera, such as *Lactobacillus*, *Dubosiella*, *Bacteroides*, *Akkermansia*, *Lachnospiraceae_NK4A136_group*, *Ruminococcaceae_UCG.014* and *Alloprevotella*, were the dominant microflora (Supplementary Figure S2C,D).

### 2.3. Xn Regulates the Diversity of Intestinal Microbiota Community

**Figure 2:**
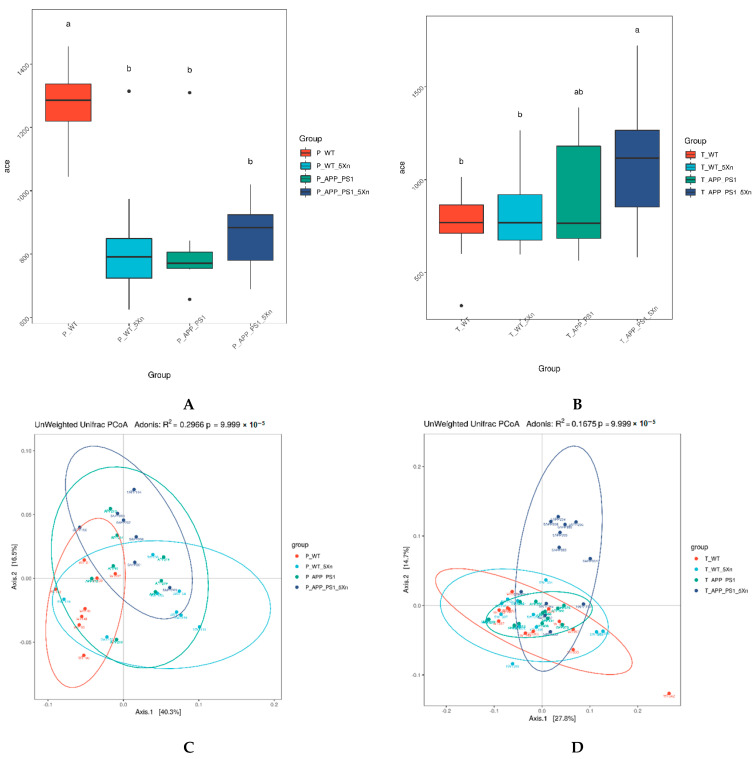

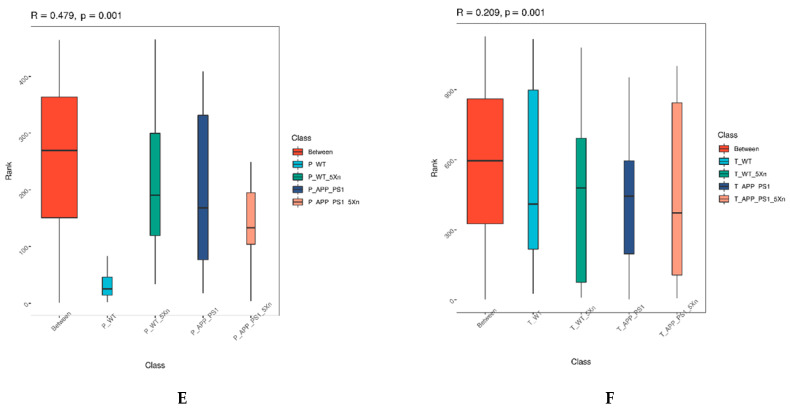
Diversity analysis among groups in the prevention and therapy experiment of Xn. (**A**,**B**) Ace index of α diversity analysis depicting the difference in microflora abundance among groups. The differences were analyzed by Tukey test or Kruskal-Wallis test–a, b represent significant difference between them; ab represents no difference with a or b. (**C**,**D**) Principal co-ordinates analysis (PCoA) of β diversity analysis depicting the difference in microflora composition among groups. The differences were analyzed by Adonis multivariate analysis of variance based on unweighted Unifrac algorithm. Each dot represents a sample, and each color represents a different grouping. The closer the two samples are, the more similar their species composition is. The horizontal and vertical coordinates represent the two principal coordinates that contribute most to the group difference. The larger the R^2^, the higher the explanation of the difference. (**E**,**F**) Anosim test used to judge whether the grouping is meaningful. R-value is between (−1, 1), and R-value is greater than 0, indicating that the difference between groups is greater than the difference within groups. R-value is less than 0, indicating that the difference within groups is greater than the difference between groups. The *p* value less than 0.05 indicates significant.

Microbiota community diversity was evaluated by α-diversity analysis, β-diversity analysis and Anosim similarity analysis [20,21]. The Ace index in the α-diversity analysis reflects the abundance of species in the community. The larger the Ace index, the higher the species diversity of samples. Species abundance in the APP/PS1 group was significantly lower when compared to that of the WT group (Figure 2A). After Xn treatment, the species abundance increased in APP/PS1 mice, both in the preventive and therapeutic experiments (Figure 2A,B). Beta diversity analysis reflects the degree of similarity in species diversity among samples from different treatments. Principal co-ordinates analysis (PCoA), an unweighted Unifrac algorithm based on a system occurrence tree, mainly finds the most important coordinates in the distance Matrix and observes the differences among groups through dimensionality reduction. The results of both prevention experiments and therapeutic experiments displayed a marked structure shift among groups after Xn treatment (Figure 2C,D). Anosim similarity analysis calculates the similarity between groups to judge whether the grouping is meaningful. The differences among groups were significantly larger than those within groups, both in the prevention experiment and therapeutic experiments (Figure 2E,F).

### 2.4. Distinct Gut Microbiome Signatures by Xn Treatment

**Figure 3:**
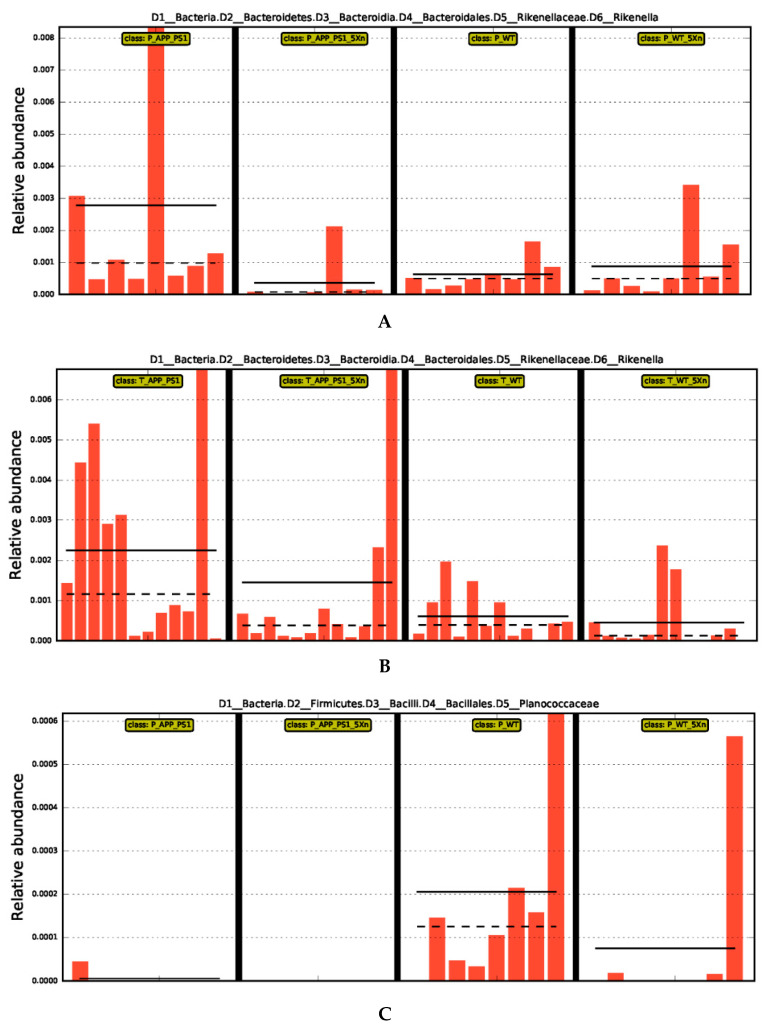

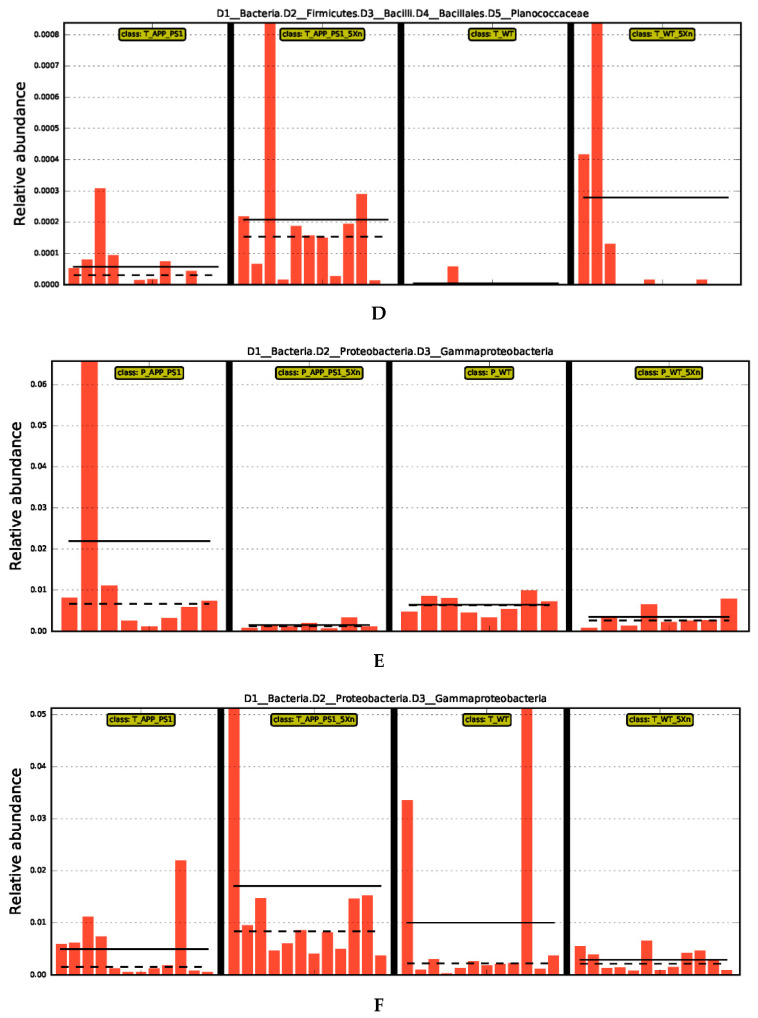
Abundance histogram of three representative differential species. (**A**,**C**,**E**) Different species in the prevention experiment. (**B**,**D**,**F**) Different species in the therapy experiment. The X-axis represents different groups, the Y-axis represents the relative abundance of different species, and the orange bars with different lengths indicate the relative abundance of different species in each sample. In the groups of each mini-graph, the solid line represents the mean value within the group, and the dashed line represents the median value. LDA score was used to assess the impact of significantly differential species. The larger the score, the bigger the impact. The pictures show different species with LDA Score greater than 2.0. LDA, Line Discriminant Analysis.

**Figure 4:**
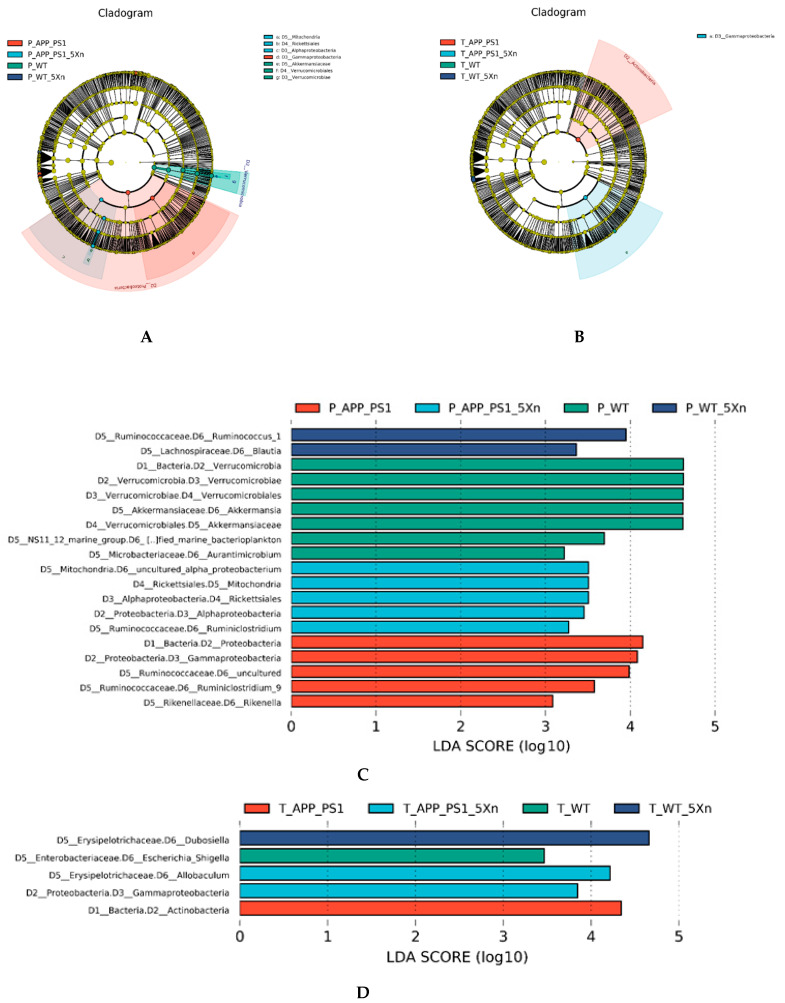
Differential species via LEfSe analysis in the prevention and therapy experiment of Xn. (**A**,**B**) Evolutionary cladogram of different species in the prevention and the therapy experiment, respectively. The circle radiating from the inside to the outside represents the level from the phylum to the species. Each small circle at different levels represents a category at that level, and the diameter of the small circle is proportional to the relative abundance. Species with no significant differences are uniformly colored yellow, and other different species are colored according to the grouping of the species. Different colors indicate different groups, and nodes of different colors indicate the microbiota play an important role in the group. (**C**,**D**) LDA score histogram of different species in the prevention and the therapy experiment, respectively. LDA score was used to assess the impact of significantly differential species. The larger the score, the bigger the impact. These pictures show different species with LDA score greater than 3.0. LDA, Line Discriminant Analysis.

To further identify the potential biomarkers with significant differences, we compared the abundance of species among groups via Line Discriminant Analysis (LDA) Effect Size (LEfSe) analysis. The LDA score was used to assess the impact of significantly differential species. At LDA Scores greater than 2.0, we found 437 differential species in the prevention experiment and 192 differential species in the therapy experiment. Ninety-two common differential species in the prevention and therapy experiment were identified (Supplementary Table S1), which shows the bacterial Domain (D1), Phylum (D2), Class (D3), Order (D4), Family (D5) and Genus (D6), respectively. Interestingly, *D1_Bacteria*, *D2_Bacteroidetes*, *D3_Bacteroidia*, *D4_Bacteroidales*, *D5_Rikenellaceae*, and *D6_Rikenella* were found in high abundance in the APP/PS1 mouse group both in the prevention and therapeutic experiments (Figure 3A,B). *D1_Bacteria*, *D2_Firmicutes*, *D3_Bacilli*, *D4_Bacillales*, and *D5_Planococcaceae* were highly enriched in WT mice in the prevention experiment and in the Xn-treated WT mice of therapeutic experiment (Figure 3C,D). *D1_Bacteria*, *D2_Proteobacteria*, and *D3_Gammaproteobacteria* were highly enriched in APP/PS1 mice in the prevention experiment and in the Xn-treated APP/PS1 mice of the therapeutic experiment (Figure 3E,F). For LDA scores > 3.0, the predominant differential microflora *D2_Proteobacteria* and *D3_Gammaproteobacteria* were still significantly enriched in the APP/PS1 mice in the prevention experiment and in the Xn-treated APP/PS1 mice of the therapeutic experiment (Figure 4).

We further analyzed the differential microflora on Family and Genus levels via Kruskal–Wallis test. On the Family level, we found 111 differential species in the prevention experiment and 94 differential species in the therapy experiment. Sixty-six common differential species in the prevention and therapy experiments were identified (Supplementary Table S2). Most of them were reduced after Xn treatment in the prevention experiment while they increased in the therapeutic experiment. Only four of them (*Alteromonadaceae*, *Nitriliruptoraceae*, *Nodosilineaceae*, *Rhodospirillaceae*) showed a similar tendency toward reduction both in the prevention and therapeutic experiments. The *uncultured* microflora showed the same increasing tendency both in the prevention and therapeutic experiments. The *uncultured_bacterium* was increased only in the Xn-treated APP/PS1 mice. On the Genus level, we also identified 80 common differential species in the prevention and therapeutic experiments (Supplementary Table S3). Three microflora *Lachnospiraceae_UCG-001*, *Nodosilinea_PCC-7104*, and *Rikenella* were reduced in the Xn-treated APP/PS1 mice both in the prevention and therapy experiments. *Prevotella_9* was increased in the Xn-treated APP/PS1 mice. Importantly, *Nodosilineaceae* was found both on the Family level and Genus level in the Xn-treated APP/PS1 mice. *Rikenellaceae* were found in two kinds of algorithms, LEfSe analysis and the Kruskal–Wallis test.

### 2.5. Xn Treatment May Regulate the Metabolic Function of the Gut Microbiome

**Figure 5:**
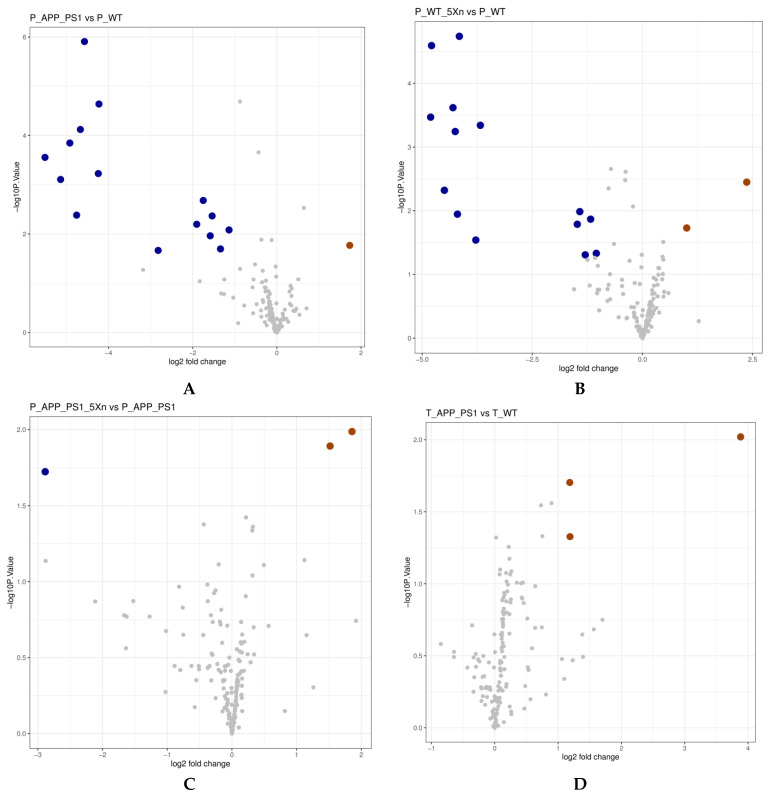

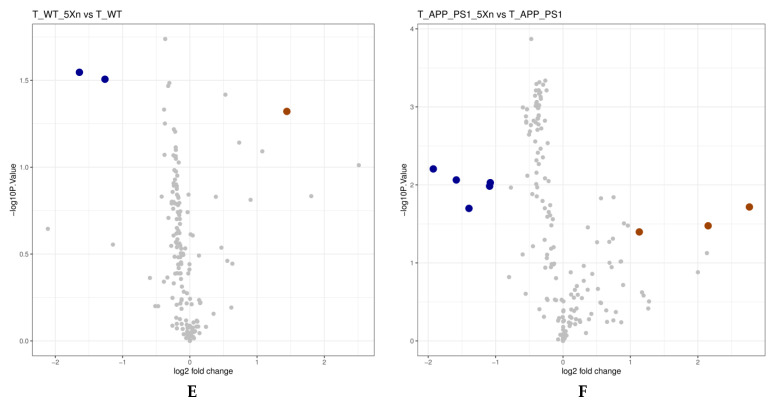
KEGG pathway volcano to predict differences in metabolic pathways of microbial communities. (**A**–**C**) KEGG pathway volcano in the prevention experiment. (**D**–**F**) KEGG pathway volcano in the therapy experiment. The X-axis represents log2 fold change, the Y-axis represents −log10 (*p* value). Here, the log2 fold change with difference multiple greater than 2 and *p* value less than 0.05 were the screening conditions. Each of these big blue dots represents a down-regulated metabolic pathway while the big purple dots represent the up-regulated metabolic pathways.

To understand the biological functions of the differential species, we performed the metabolic function prediction analysis in the Kyoto Encyclopedia of Genes and Genomes (KEGG) pathway database via Phylogenetic Investigation of Communities by Reconstruction of Unobserved States (PICRUSt) software. We found four up-regulated and 17 down-regulated metabolic pathways in the prevention experiment (Figure 5A and Table 1). Seven metabolic pathways were up-regulated, and five metabolic pathways were down-regulated in the therapeutic experiment (Figure 5B and Table 2). Importantly, four common pathways were found in both the prevention and therapeutic experiments. The photosynthesis pathway and caprolactam degradation pathway were down-regulated in the prevention experiment while up-regulated in the therapeutic experiment. The penicillin and cephalosporin biosynthesis pathway and the atrazine degradation pathway were up-regulated after Xn treatment in APP/PS1 mice of the prevention experiment while down-regulated in the therapeutic experiment.

## 3. Discussion

Recently, great progress had been made in understanding the relationship between gut microbiota and AD. With the preliminary success in AD therapy of oral treatment with the marine algae-derived oral oligosaccharide sodium oligomannate (GV-971), increasing attention has been paid to the possibility that other natural compounds may have efficacy in the prevention and treatment of AD [22,23]. CA-30, an oligosaccharide fraction derived from *Liuwei Dihuang* (LW) used in Traditional Chinese Medicine, improves cognitive function via the gut microbiota in the senescence-accelerated mouse prone 8 (SAMP8) strain [24]. A 70% ethanol extract of *Tetragonia tetragonioides* Kuntze (TTK) enhanced memory function and the intestinal microbiome in Aβ-infused rats [25]. However, methods and mechanisms to optimize gut microbiota for the alleviation of AD are still far from being understood. In our study, we identified differential gut microbiota related to AD after Xn treatment via 16S rDNA section sequencing of APP/PS1 vs WT mice. Behavioral results showed that Xn could prevent and slow down the development of cognitive impairment in these transgenic animals. This was consistent with results reported in previous work [26]. In brief, Xn treatment restored the normal composition and function of gut microbiota.

The normal intestinal microflora is mainly composed of *Bifidobacterium*, *Bacteroides*, *Enterococcus*, *Lactobacillus*, *Fusobacterium*, *Eubacterium* and *Proteus* species. Their balance plays a key role in maintaining bodily homeostasis. Changes in the composition and/or abundance of gut microbiota have been demonstrated in various diseases. In the present study, the abundance of gut microbiota in APP/PS1 mice was different from that in WT mice, and prolonged oral Xn treatment restored the abundance in the former to a certain extent, as shown by significant differences in the diversity analysis. However, the Ace index in the α-diversity analysis of the Xn therapeutic experiment was not obviously significant, which may have been related to individual animal differences in the group.

Previous studies [27] have shown that Xn has strong antibacterial activity against *Staphylococcus aureus*, *Listeria monocytogene* and other Gram-positive bacteria, as well as acid-fast bacteria and fungi. In this study, we found that the regulatory abundance and trends of gut microbiota by Xn treatment were different both in the preventive and therapeutic experiments, and in most cases were reversed. This is an interesting case that deserves further consideration. It may be that during the aging process [28] or other intervention factors such as voluntary exercise [29], the composition and abundance of bacteria related to AD changed correspondingly. Others have found that the presence of several intestinal bacterial families, including *Rikenellaceae*, significantly discriminate patients with schizophrenia (SCZ) from healthy controls based on the abundance of differential microbiota via a stepwise regression analysis [30]. Here we report some previously unidentified microbial signatures associated with AD. We also found that *Rikenellaceae* was related with AD after Xn treatment. *Nodosilineaceae* is a new strain related to AD that is first reported here.

Accumulating evidence shows that metabolic changes associated with gut microbiota are important factors relating to various diseases. Here, we found that Xn was able to reshape the entire intestinal microbial structure of APP/PS1 mice, involving the Penicillin and cephalosporin biosynthesis pathway and the Atrazine degradation pathways. The latter is a useful pathway mainly for the degradation of *Noncardiac* in wastewater [31]. In the gut, the Atrazine degradation pathway may be used primarily to clear excess or unwanted bacterial.

To our knowledge, this is the first study to investigate whether Xn can regulate the composition of gut microbiota in a mouse AD model via 16S rDNA sequencing technique and PICRUSt forecasting software. Some shortcomings existed in the study. Firstly, the effects of Xn treatment were not obviously significant because of bioavailability when administered by the oral route [32,33]. Secondly, dysfunction of learning and memory in APP/PS1 mice is apparent at 6–8 months of age, such that Xn treatment may have better results if continued for a longer time period. Thirdly, behavioral evidence alone is insufficiently persuasive of a preventive or therapeutic effect of Xn treatment. This should be supplemented by neuropathological indices of AD, such as the deposition of Aβ and Tau proteins. Lastly, the 16S rDNA sequencing technique, while effective in identifying specific genera, cannot accurately identify microflora species. More advanced techniques, such as third-generation sequencing, should be adapted to verify the identity of bacterial species.

## 4. Materials and Methods

### 4.1. APP/PS1 Mice and Xn Administration

APPswe/PS1de9 double-transgenic mice (APP/PS1 mice) and C57 mice of the same age and genetic background were used in our study. Animal treatment and care were performed in accordance with the Principles of Laboratory Animal Care (NIH publication No. 85-23, revised in 1985) and the Regulations for Animal Care and Use from the Committee of the Experimental Animal Center at Shenzhen Center for Disease Control and Prevention in Shenzhen, Guangdong Province, China. This animal study was approved by Shenzhen Center for Disease Control and Prevention Ethics Committee (NO.2019029). Efforts were made to minimize animal suffering and reduce the number of mice used for experiments. Food and water were provided and freely available.

**Figure 6:**
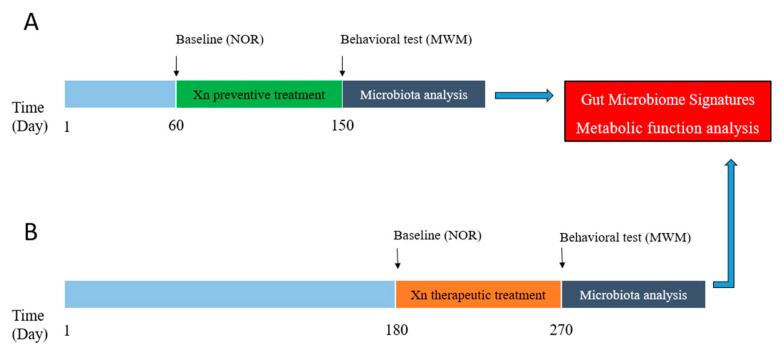
Schematic diagram of the experimental design. (**A**) Diagram depicting the prevention experiment. Before xanthohumol (Xn) administration at age of 60 days, novel object recognition (NOR) test in the baseline was detected. (**B**) Diagram depicting the therapy experiment. Before Xn administration at age of 180 day, NOR test in the baseline was also detected. Morris water maze (MWM) test and fecal samples for microbiota were detected after Xn administration for 3 months both in the prevention and therapy experiments. NOR, novel object recognition test. MWM, Morris water maze test.

Xn intervention experiments included preventive and therapeutic treatment strategies according to the pathological features of APP/PS1 mice (Figure 6). Preventive experiments were performed using 2-month-old mice (at 60 days of age), while therapeutic experiments employed 6-month-old animals (at 180 days of age), that is, prior to and following the expression of cognitive deficits. The preventive and therapeutic experiments comprised four groups of equal numbers of male and female mice consisting of 9–11 and 11–14 animals, respectively: (a) wild-type (WT) group were dosed by gavage every other day for 3 months with corn oil (0.1 mL/10 g body weight), alone; (b) the WT_5Xn group with 5 mg/kg Xn; (c) the APP/PS1 group with corn oil; and (d) the APP/PS1_5Xn group with 5 mg/kg Xn. The mice were treated with Xn or corn oil by oral gavage every other day for 90 days. Before administration, cognitive function was determined using the novel object recognition (NOR) test [34]. One day prior to NOR testing, animals were transferred from their cages to the behavioral laboratory, which provided a stable, quiet environment. Testing was conducted between 8:00 a.m. and 3:00 p.m. After testing, the test area (plastic box) was cleaned with alcohol solution to remove urine, feces and associated odor.

### 4.2. Behavioral Tests

The NOR test evaluates short-term memory function based on the length of time that animals take to explore familiar versus unfamiliar objects. On day one, mice were individually allowed to explore freely in the plastic box without objects for 5 min in order to adapt to the environment. On day 2 (i.e., 24 h after day 1), the animals were individually placed in the same box with two identical objects for 5 min. The mouse was then removed from the box to rest for one hour. One of the objects was then replaced in the same position with a new object of the same material, color and size, but different in shape. The mouse was then returned to the box and allowed to explore freely for 5 min. The time taken to explore new and old objects was recorded by an animal behavior video analysis system (Xeye Aba V3.2, MacroAmbition S&T Development Co. Ltd., Beijing, China). The exploratory time (minimum of 10s) was taken as the time the mouth, nose or forepaw was in direct contact with the object or when the animal was close to the object within a radius of ≤2 cm. The recognition index (new object exploration time/total exploration time × 100%) was used to determine the recognition coefficient.

After Xn treatment, the Morris water maze (MWM) was used to test spatial learning and memory. The MWM test included the navigation experiment and the space search experiment [35]. The experimental device consisted of a circular pool with a diameter of 120 cm and a height of 40 cm. The water temperature was maintained at 21 ± 2 °C. The pool was divided into four equal quadrants with four different shapes on the pool wall. A white circular platform with a diameter of 12 cm and a height of 24 cm was placed 30 cm away from the pool wall in the target quadrant, and the water level covered the platform by 1–2 cm. An animal behavior video analysis system (Xeye Aba V3.2, Beijing MacroAmbition S&T Development Co., LTD, Beijing, China) was used to collect and process relevant data. The navigation experiment lasted for 5 days. Each mouse was trained 4 times a day for 60 s per session. The platform residence time was set at 3 s. A quadrant was randomly selected. The animal was placed into the water facing the wall of the pond, and the time taken to find the platform was recorded. If the platform was not found within 60 s, the animal was led to the platform where it was allowed to stay for 15 s. Each mouse was trained a total of 20 times, and the average escape latency of 4 times per day was calculated. On Day 7, the spatial search experiment was used to measure memory ability. The platform was removed, and the mice were placed in the water to measure their time in the target quadrant, probe time, swimming path, swimming speed, and the number of times they crossed the platform over a 120 s period.

### 4.3. Stool Sample Collection and Preservation

The operating table, laboratory instruments and consumables were all sterilized to maintain aseptic conditions. Mice were anesthetized with 10% chloral hydrate (10 mL/kg) administered intraperitoneally, after which 2–4 pieces of fresh stool were removed from the end of the colon. The whole intestine was taken out under aseptic conditions; the middle and upper segment of the rectum was cut with aseptic scissors, and fecal samples (1–2 g) extruded in a 1.5 mL EP tube (aseptic tube). The samples were immediately immersed in liquid nitrogen and stored at −80 °C or later use.

### 4.4. 16S rDNA Sequencing

Microbial diversity analysis was performed by 16S rDNA section sequencing. The whole process included: sample DNA extraction, PCR amplification and purification, library preparation, database building, taxonomic annotation and biological interpretation. Total genome DNA from samples was extracted using the cetyltrimethylammonium bromide (CTAB)/sodium dodecyl sulfate (SDS) method. DNA concentration and purity were monitored on 1% agarose gels. According to the concentration, DNA was diluted to 1 ng/μL using sterile water. 16S V3-V4 regions were amplified using a specific primer with the barcode. 341F primer sequence (5′–3′): CCTAYGGGRBGCASCAG, 806R primer sequence (5′–3′): GGACTACNNGGGTATCTAAT. All PCR reactions were carried out with Phusion^®^ High-Fidelity PCR Master Mix (New England Biolabs, Ipswich, MA, USA). PCR products were mixed with the same volume of 1X loading buffer (contained SYB green) and subjected to electrophoresis on a 2% agarose gel. Samples with a bright main strip between 400–450 bp were chosen for further experiments.

PCR products were then mixed in equidensity ratios. Mixed PCR products were purified with Qiagen Gel Extraction Kit (Qiagen, Hilden, Germany). Sequencing libraries were generated using the TruSeq^®^ DNA PCR-Free Sample Preparation Kit (Illumina, San Diego, CA, USA) following the manufacturer′s recommendations, and index codes were added. The library quality was assessed on the Qubit@ 2.0 Fluorometer (Thermo Fisher Scientific, Waltham, MA, USA) and Agilent Bioanalyzer 2100 system (Agilent, Santa Clara, CA, USA). The library was sequenced on an Illumina HiSeq2500 platform, and 250 bp paired-end reads were generated. The original reads were cut and filtered to form Tags, and the target fragments were clustered to form Operational Taxonomy Units (OTUs). According to the total OTUs, the common and specific OTUs were analyzed statistically, and then the species were annotated to obtain information of the community composition at different levels of each sample.

### 4.5. Data Processing and Bioinformatics

16S microbial diversity information analysis included three parts: data processing and quality control, basic analysis and advanced analysis. Data processing and quality control were achieved mainly through the patchwork filtering and data evaluation of the original sequenced reads and access to follow-up analysis of the available high-quality clean tags. The basic analysis involved clustering OTU on the processed tags, followed by performing species annotation and diversity analysis.

Basic analysis included OTU cluster analysis, species taxonomy, Alpha diversity and Beta diversity analysis. OTU cluster analysis was performed via ultrafast sequence analysis (Usearch) software version 11.0.667 (http://www.drive5.com/usearch/) (access on 3 January 2022) according to a 97% similarity sequence. Species composition annotation was based on the results of taxonomic analysis to compare the community structure differences of samples. The species composition and abundance of different samples were plotted on bar graphs at the level of phylum, class, order, family and genus. Diversity analysis was mainly used to assess sample abundance and analyzed using R language version 4.0.5 in Rstudio using the Linux platform to visualize data mapping. Ace index in Alpha diversity and principal co-ordinates analysis (PCoA) in Beta diversity was used to compare differences among groups.

Advanced analysis included significant difference analysis and functional prediction analysis. Anosim similarity analysis is a non-parametric test to determine whether differences among groups are significantly greater than differences within groups. Line Discriminant Analysis (LDA) Effect Size (LEfSe) analysis is used to calculate significant biological differences among groups. LDA (log10) score > 2 is widely used as a potential biomarker. The metabolic function of microorganism was predicted and evaluated by Phylogenetic Investigation of Communities by Reconstruction of Unobserved States (PICRUSt) software.

### 4.6. Statistical Analysis

SPSS version 25.0 was used to analyze the data. One-way ANOVA was used to compare the mean between groups followed with the LSD-t test. Repeated measures ANOVA was used in the MWM test. Graphs were drawn via Graphpad Prism version 8 and R language version 4.0.5, using $\bar{X}$ ± *SEM* to represent the data. *p* < 0.05 has statistical significance.

## 5. Conclusions

We used 16S rDNA sequencing to analyze the effect of oral Xn, a prenylated flavonoid from hops (*Humulus lupulus* L.), on the gut microbiome and its metabolic pathways in APP/PS1 mice. Our findings demonstrate the protective role of Xn in regulating gut microbiome composition and metabolism in the development of this animal model of AD. Based on these findings, we propose further studies to determine the potential role of this natural compound in the prevention and therapy of human AD. Xn as a dietary supplement has already completed a phase-I clinical trial [36,37]. It is reasonable to establish an AD-related specific bacterial strain library for future targeted study with Xn and other compounds.

## Figures and Tables

**Table 1:** Differential KEGG pathways predicted via PICRUSt software in the Xn prevention experiment.

Number	Description	P_APP_PS1_vs_P_WT			P_WT_5Xn_vs_P_WT			P_APP_PS1_5Xn_vs_P_APP_PS1		
		log2 (Fold Change)	*p* Value	Up/Down	log2 (Fold Change)	*p* Value	Up/Down	log2 (Fold Change)	*p* Value	Up/Down
ko05010	Alzheimer′s disease	1.732027242	0.017025339	up	2.366161715	0.003558436	up			
ko04974	Protein digestion and absorption				1.00459934	0.018638107	up			
ko00830	Retinol metabolism	−4.572428141	1.24 × 10^−5^	down	−4.157153572	1.83 × 10^−5^	down			
ko04142	Lysosome	−4.227876257	2.30 × 10^−5^	down	−3.679825761	0.000455767	down			
ko00980	Metabolism of xenobiotics by cytochrome P450	−4.66899817	7.55 × 10^−5^	down	−4.787486299	2.55 × 10^−5^	down			
ko00363	Bisphenol degradation	−4.919555706	0.000142318	down	−4.808225969	0.000339327	down			
ko00195	Photosynthesis	−5.509890045	0.000278555	down	−4.495865401	0.00477363	down			
ko00642	Ethylbenzene degradation	−4.243304316	0.000594088	down	−4.249603903	0.000570651	down			
ko00523	Polyketide sugar unit biosynthesis	−5.139057953	0.000781219	down	−4.199953052	0.011321054	down			
ko00311	Penicillin and cephalosporin biosynthesis	−1.749057294	0.002085877	down	−1.299383141	0.049212447	down	1.517322773	0.012792112	up
ko00253	Tetracycline biosynthesis	−4.758378649	0.004146843	down	−3.783153775	0.028842448	down			
ko00364	Fluorobenzoate degradation	−1.537476491	0.004302729	down	−1.422606062	0.010323156	down			
ko00791	Atrazine degradation	−1.904490251	0.006353534	down				1.85619649	0.010297892	up
ko00906	Carotenoid biosynthesis	−1.136194803	0.008280216	down	−1.047940043	0.046641794	down			
ko05200	Pathways in cancer	−1.582997558	0.010892181	down						
ko00930	Caprolactam degradation	−1.339589301	0.020127255	down	−1.479654328	0.016294803	down			
ko00903	Limonene and pinene degradation	−2.82113561	0.021578285	down	−4.301375008	0.000240924	down			
ko00643	Styrene degradation				−1.177109587	0.013561769	down			
ko00622	Xylene degradation							−2.887030853	0.018878669	down

Differential pathways with log2 fold change > 1. Black boldface words indicate representative KEGG pathways. PICRUSt, Phylogenetic Investigation of Communities by Reconstruction of Unobserved States.

**Table 2:** Differential KEGG pathways predicted via PICRUSt software in the Xn therapy experiment.

Number	Description	T_APP_PS1_vs_T_WT			T_WT_5Xn_vs_T_WT			T_APP_PS1_5Xn_vs_T_APP_PS1		
		log2 (Fold Change)	*p* Value	Up/Down	log2 (Fold Change)	*p* Value	Up/Down	log2 (Fold Change)	*p* Value	Up/Down
ko00195	Photosynthesis	3.882765365	0.009566875	up						
ko00930	Caprolactam degradation	1.184272747	0.019807356	up						
ko05150	Staphylococcus aureus infection	1.188139551	0.047056443	up				−1.583094991	0.008640976	down
ko00472	D-Arginine and D-ornithine metabolism				1.443191914	0.047738796	up			
ko00791	Atrazine degradation				−1.642519851	0.028425143	down	−1.924538818	0.00624072	down
ko00311	Penicillin and cephalosporin biosynthesis				−1.262387166	0.031172616	down	−1.395234979	0.020013002	down
ko00623	Toluene degradation							2.764724413	0.019188937	up
ko04142	Lysosome							2.152419965	0.033435027	up
ko00364	Fluorobenzoate degradation							1.131389736	0.040093241	up
ko00621	Dioxin degradation							−1.078840557	0.009368244	down
ko02060	Phosphotransferase system (PTS)							−1.088900276	0.010383481	down

Differential pathways with log2 fold change > 1. Black boldface words indicate representative KEGG pathway. PICRUSt, Phylogenetic Investigation of Communities by Reconstruction of Unobserved States.

## Data Availability

The data presented in this study were available on request from the corresponding author.

## Supplementary Materials

The following are available online, Figure S1: Baseline data of NOR test in the prevention and therapeutic experiments using Xn, Figure S2: Species taxonomy comparison of intestinal microbiome on Family and Genus levels in the prevention and therapeutic experiments with Xn, Table S1: Ninety-two common differential species in the prevention and therapeutic experiments via LEfSe analysis, Table S2: Abundance comparison of differential bacteria on the Family level in the prevention and therapeutic experiments, Table S3: Abundance comparison of differential bacteria on the Genus level in the prevention and therapeutic experiments.
