# Supplementary material for: Natural Dietary Compound Xanthohumol Regulates the Gut Microbiota and Its Metabolic Profile in a Mouse Model of Alzheimer’s Disease
Source: Molecules. 2022 Feb 14;27(4):1281. doi: 10.3390/molecules27041281 (PMC8880053; doi:10.3390/molecules27041281)
Supplement: Supplementary file 1 [file molecules-27-01281-s001.zip › molecules-1564368-supplementary.pdf]

Supplementary Materials

Natural Dietary Compound Xanthohumol Regulates the Gut Microbiota and Its Metabolic Profile in a Mouse Model of Alzheimer's Disease

Wei Liu, Kaiwu He, Desheng Wu, Li Zhou, Guowei Li, Zequn Lin, Xifei Yang, Jianjun Liu, Maggie Pui Man Hoi

Contents

Figure S1 Baseline data of NOR test in the prevention and therapeutic experiments using Xn.

Figure S2 Species taxonomy comparison of intestinal microbiome on Family and Genus levels in the prevention and therapeutic experiments with Xn.

Table S1 Ninety-two common differential species in the prevention and therapeutic experiments via LEfSe analysis.

Table S2 Abundance comparison of differential bacteria on the Family level in the prevention and therapeutic experiments.

Table S3 Abundance comparison of differential bacteria on the Genus level in the prevention and therapeutic experiments.

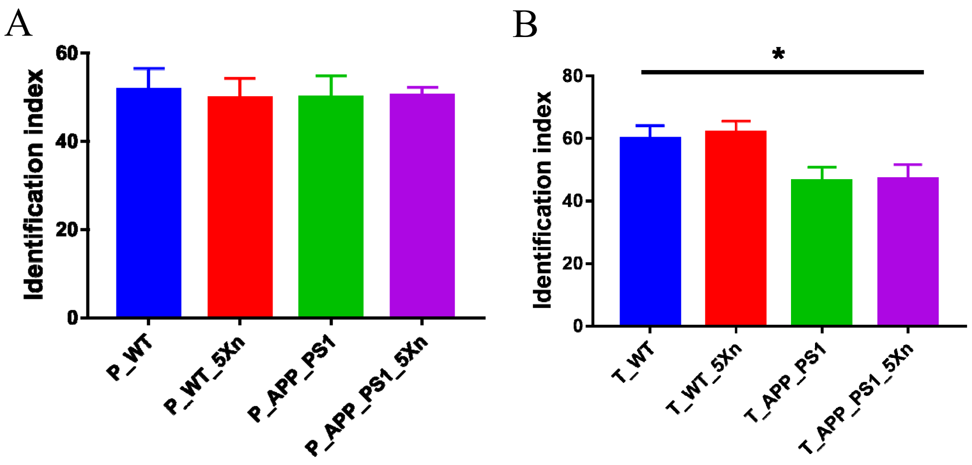

Figure S1 Baseline data from the NOR test in the prevention and therapeutic experiments using Xn. (A) NOR test in the prevention experiment (n = 7-11 per group). No difference was found in the baseline (F=0.04, P=0.989). (B) NOR test in the therapeutic experiment (n = 10-14 per group). Significant difference was found in the baseline (F = 3.74, p = 0.0183). NOR, novel object recognition test. The baseline data from the NOR test were analyzed by one-way ANOVA. Error bar, SEM.

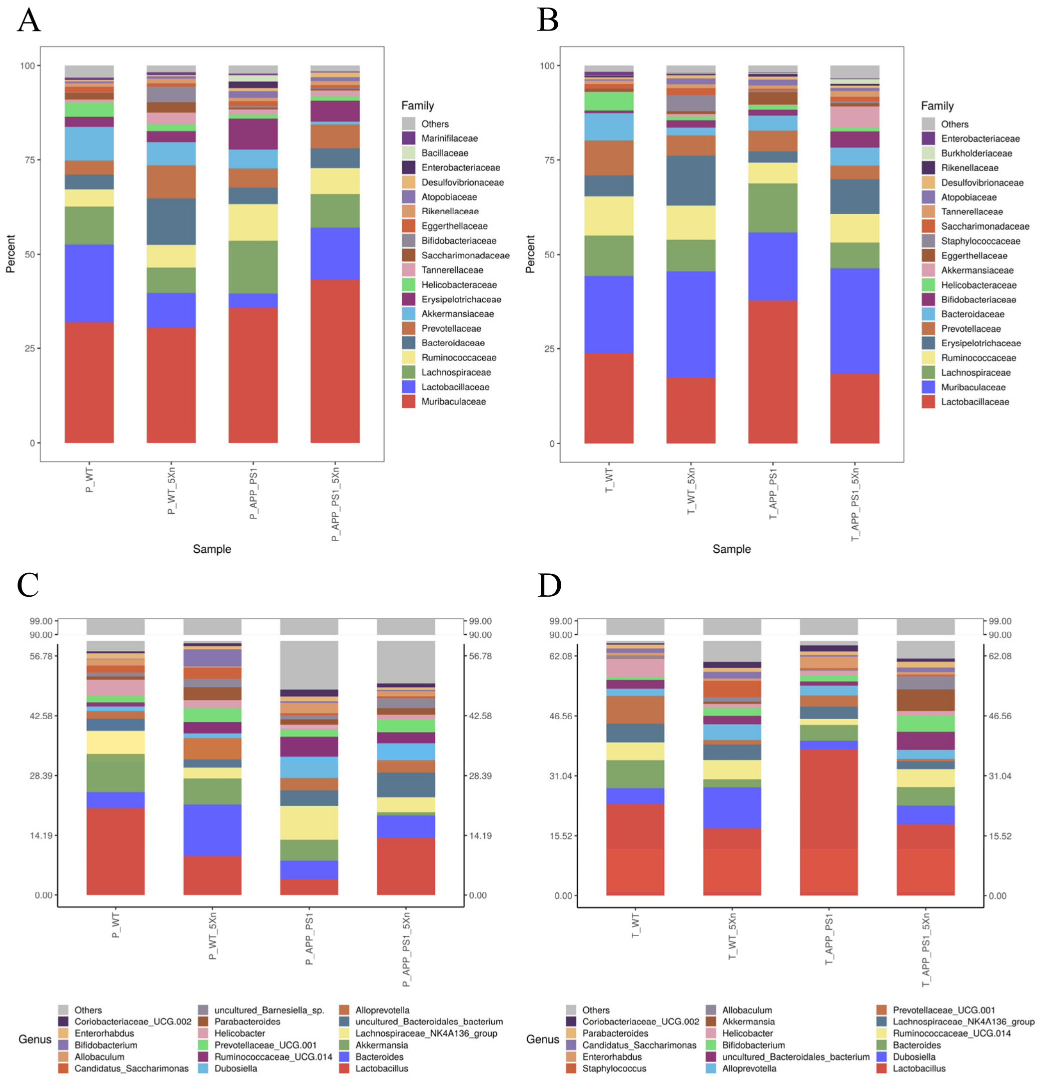

Figure S2 Species taxonomy comparison of intestinal microbiome on Family and Genus levels in the prevention and therapeutic experiments with Xn. (**A,C**) Species composition and abundance analysis in the prevention experiment (*n* = 7-8 per group). (**B,D**) Species composition and abundance analysis in the therapeutic experiment (*n* =12 per group). Different colors of the columns indicate different species. The length of the columns indicates the relative abundance of the species.

Table S1 Ninety-two common differential species in the prevention and therapeutic experiments via LEfSe analysis.

Different Species	Prevention Experiment				Therapy Experiment			
	Relative Abundance	Group	LDA	*p*	Relative Abundance	Group	LDA	*p*
D1__Bacteria.D2__Actinobacteria.D3__Acidimicrobiia	3.048704018	P_WT	2.76914427	0.000332677	2.969026709	T_APP_PS1_5Xn	2.678142775	0.001313013
D1__Bacteria.D2__Actinobacteria.D3__Acidimicrobiia.D4__Microtrichales	3.044851296	P_WT	2.766523768	0.000164917	2.94561858	T_APP_PS1_5Xn	2.652557189	0.001457651
D1__Bacteria.D2__Actinobacteria.D3__Acidimicrobiia.D4__Microtrichales.D5__Ilumatobacteraceae	2.966081954	P_WT	2.686010091	9.55×10^{-5}	2.854020132	T_APP_PS1_5Xn	2.559676669	0.001457651
D1__Bacteria.D2__Actinobacteria.D3__Acidimicrobiia.D4__Microtrichales.D5__Ilumatobacteraceae.D6__uncultured	1.934759374			0.006074663	1.628779518			0.003091452
D1__Bacteria.D2__Actinobacteria.D3__Acidimicrobiia.D4__Microtrichales.D5__Microtrichaceae	1.534198621			0.008053849	1.833581771			0.006866718
D1__Bacteria.D2__Actinobacteria.D3__Acidimicrobiia.D4__Microtrichales.D5__Microtrichaceae.D6__Sva0996_marine_group	1.214402197			0.033801621	1.691314522			0.007761456
D1__Bacteria.D2__Actinobacteria.D3__Acidimicrobiia.D4__Microtrichales.D5__uncultured	1.99046404			0.000698642	1.919978996			0.012736314
D1__Bacteria.D2__Actinobacteria.D3__Acidimicrobiia.D4__Microtrichales.D5__uncultured.D6__metagenome	1.82418793			0.000861258	1.872409677			0.003874887
D1__Bacteria.D2__Actinobacteria.D3__Actinobacteria.D4__Corynebacteriales	2.179748085			0.012529197	2.45567934	T_APP_PS1	2.140633491	0.019423178
D1__Bacteria.D2__Actinobacteria.D3__Actinobacteria.D4__Corynebacteriales.D5__Mycobacteriaceae	2.088117768			0.002284315	1.947337743			0.008899185
D1__Bacteria.D2__Actinobacteria.D3__Actinobacteria.D4__Corynebacteriales.D5__Mycobacteriaceae.D6__Mycobacterium	2.088117768			0.002284315	1.947337743			0.008899185
D1__Bacteria.D2__Actinobacteria.D3__Actinobacteria.D4__Micrococcales.D5__Microbacteriaceae.D6__Candidatus_Aquiluna	2.573450444	P_WT	2.28438957	0.000147682	2.555093182	T_APP_PS1_5Xn	2.269665678	0.002544355
D1__Bacteria.D2__Actinobacteria.D3__Actinobacteria.D4__PeM15	2.488257536	P_WT	2.198336881	0.000120269	2.59019525	T_APP_PS1_5Xn	2.279306043	0.000922367
D1__Bacteria.D2__Actinobacteria.D3__Actinobacteria.D4__PeM15.D5__metagenome	2.332596926	P_WT	2.059576939	0.000150807	2.433292362	T_APP_PS1_5Xn	2.128989119	0.000731003
D1__Bacteria.D2__Actinobacteria.D3__Actinobacteria.D4__PeM15.D5__metagenome.D6__metagenome	2.332596926	P_WT	2.058427205	0.000150807	2.433292362	T_APP_PS1_5Xn	2.1290613	0.000731003
D1__Bacteria.D2__Actinobacteria.D3__Actinobacteria.D4__PeM15.D5__uncultured_bacterium	1.967143836			0.000185142	2.071949795			0.001157323
D1__Bacteria.D2__Actinobacteria.D3__Actinobacteria.D4__PeM15.D5__uncultured_bacterium.D6__uncultured_bacterium	1.967143836			0.000185142	2.071949795			0.001157323
D1__Bacteria.D2__Actinobacteria.D3__Thermoleophilia	2.109651559	P_WT	2.015179734	0.001188053	2.624350577	T_APP_PS1_5Xn	2.421815196	0.008732333
D1__Bacteria.D2__Actinobacteria.D3__Thermoleophilia.D4__Solirubrobacterales	1.984070535	P_WT	2.081223232	0.001451998	2.150007691			0.004483976
D1__Bacteria.D2__Actinobacteria.D3__Thermoleophilia.D4__Solirubrobacterales.D5__67_14	1.77298476			0.001273353	1.920994678			0.004483976

D1__Bacteria.D2__Actinobacteria.D3__Thermoleophilia.D4__Solirubrobacterales.D5__67_14.D6__uncultured_bacterium	1.524675144			0.001888883	1.401319557			0.029588168
D1__Bacteria.D2__Bacteroidetes.D3__Bacteroidia.D4__Bacteroidales.D5__Barnesiellaceae	1.462885885			0.020581901	2.163004549			0.007518415
D1__Bacteria.D2__Bacteroidetes.D3__Bacteroidia.D4__Bacteroidales.D5__Barnesiellaceae.D6__uncultured	1.462885885			0.020581901	1.668827796			0.018343846
**D1__Bacteria.D2__Bacteroidetes.D3__Bacteroidia.D4__Bacteroidales.D5__Rikenellaceae.D6__Rikenella**	**3.444157657**	**P_APP_PS1**	**3.084689205**	**0.018362202**	**3.352872674**	**T_APP_PS1**	**2.994826976**	**0.030077956**
D1__Bacteria.D2__Bacteroidetes.D3__Bacteroidia.D4__Bacteroidetes_VC2_1_Bac22	1.926144545			7.44×10^{-5}	2.353768065			0.00022668
D1__Bacteria.D2__Bacteroidetes.D3__Bacteroidia.D4__Bacteroidetes_VC2_1_Bac22.D5__uncultured_Bacteroidetes_bacterium	1.835998776			0.000453238	2.348409688			0.000202454
D1__Bacteria.D2__Bacteroidetes.D3__Bacteroidia.D4__Bacteroidetes_VC2_1_Bac22.D5__uncultured_Bacteroidetes_bacterium.D6__uncultured_Bacteroidetes_bacterium	1.835998776			0.000453238	2.348409688			0.000202454
D1__Bacteria.D2__Bacteroidetes.D3__Bacteroidia.D4__Chitinophagales	3.078072338	P_WT	2.789523963	0.000164917	3.00779975	T_APP_PS1_5Xn	2.708914163	0.00641969
D1__Bacteria.D2__Bacteroidetes.D3__Bacteroidia.D4__Chitinophagales.D5__Chitinophagaceae	2.617262691	P_WT	2.322223506	0.000188266	2.545791491	T_APP_PS1_5Xn	2.214695145	0.009713251
D1__Bacteria.D2__Bacteroidetes.D3__Bacteroidia.D4__Chitinophagales.D5__Saprospiraceae	2.888822866	P_WT	2.605643005	0.000131568	2.794841163	T_APP_PS1_5Xn	2.51325456	0.000726278
D1__Bacteria.D2__Bacteroidetes.D3__Bacteroidia.D4__Chitinophagales.D5__Saprospiraceae.D6__uncultured	2.691574309	P_WT	2.406388822	0.000164917	2.67705156	T_APP_PS1_5Xn	2.390397453	0.001782606
D1__Bacteria.D2__Bacteroidetes.D3__Bacteroidia.D4__Cytophagales	2.840367926	P_WT	2.542410491	0.000380283	2.592715569	T_APP_PS1_5Xn	2.25699037	0.007102567
D1__Bacteria.D2__Bacteroidetes.D3__Bacteroidia.D4__Cytophagales.D5__Cyclobacteriaceae	2.439735644	P_WT	2.150188165	0.000150807	2.444513193	T_APP_PS1_5Xn	2.163813777	0.001020201
D1__Bacteria.D2__Bacteroidetes.D3__Bacteroidia.D4__Cytophagales.D5__Cyclobacteriaceae.D6__Algoriphagus	2.190891163			0.000188266	2.255170187			0.001046176
D1__Bacteria.D2__Bacteroidetes.D3__Bacteroidia.D4__Cytophagales.D5__Spirosomaceae	2.007759639			0.008587382	1.829355584			0.013525018
D1__Bacteria.D2__Bacteroidetes.D3__Bacteroidia.D4__Cytophagales.D5__Spirosomaceae.D6__Lacihabitans	1.298982341			0.026591777	1.33560638			0.005249029
D1__Bacteria.D2__Bacteroidetes.D3__Bacteroidia.D4__Flavobacteriales.D5__Crocinitomicaceae	2.189900993			0.000211824	2.001250264			0.00282806
D1__Bacteria.D2__Bacteroidetes.D3__Bacteroidia.D4__Flavobacteriales.D5__Cryomorphaceae.D6__NS10_marine_group	1.51338307			0.008053849	1.843696762			0.00120839
D1__Bacteria.D2__Bacteroidetes.D3__Bacteroidia.D4__Sphingobacteriales	2.944226285	P_WT	2.652192902	0.000126849	2.889856197	T_APP_PS1_5Xn	2.621860222	0.004133875
D1__Bacteria.D2__Bacteroidetes.D3__Bacteroidia.D4__Sphingobacteriales.D5__AKYH767	2.150143266			7.44×10^{-5}	2.094657464			0.00065909
D1__Bacteria.D2__Bacteroidetes.D3__Bacteroidia.D4__Sphingobacteriales.D5__AKYH767.D6__uncultured_bacterium	2.150143266			7.44×10^{-5}	2.085171219			0.000729701
D1__Bacteria.D2__Bacteroidetes.D3__Bacteroidia.D4__Sphingobacteriales.D5__env_OPS_17	1.624228883			0.000470142	1.706757578			0.000916083
D1__Bacteria.D2__Bacteroidetes.D3__Bacteroidia.D4__Sphingobacteriales.D5__env_OPS_17.D6__uncultured_bacterium	1.624228883			0.000470142	1.681497881			0.000916083
D1__Bacteria.D2__Bacteroidetes.D3__Ignavibacteria.D4__OPB56.D5__metagenome	2.656992993	P_WT	2.375656118	0.000356732	2.655022663	T_APP_PS1_5Xn	2.390421715	0.000680001
D1__Bacteria.D2__Bacteroidetes.D3__Ignavibacteria.D4__OPB56.D5__metagenome.D6__metagenome	2.656992993	P_WT	2.37572317	0.000356732	2.655022663	T_APP_PS1_5Xn	2.390421951	0.000680001

D1__Bacteria.D2__Bacteroidetes.D3__Rhodothermia	2.169259412			3.74×10^{-5}	2.171596243			0.000632031
D1__Bacteria.D2__Chloroflexi	2.597746366	P_WT	2.311359739	6.02×10^{-5}	2.626351345	T_APP_PS1_5Xn	2.396870814	0.002529705
D1__Bacteria.D2__Chloroflexi.D3__Chloroflexia.D4__Chloroflexales	2.20625423			4.72×10^{-5}	1.933674538			0.004640837
D1__Bacteria.D2__Chloroflexi.D3__Chloroflexia.D4__Chloroflexales.D5__Roseiflexaceae	2.119647002			2.60×10^{-5}	1.827035361			0.001353364
D1__Bacteria.D2__Chloroflexi.D3__Chloroflexia.D4__Chloroflexales.D5__Roseiflexaceae.D6__uncultured	2.119647002			2.60×10^{-5}	1.827035361			0.001353364
D1__Bacteria.D2__Chloroflexi.D3__Chloroflexia.D4__Thermomicrobiales	1.609634554			0.000861258	1.170711391			0.01195591
D1__Bacteria.D2__Chloroflexi.D3__Chloroflexia.D4__Thermomicrobiales.D5__JG30_KF_CM45	1.539004708			0.001049695	1.170711391			0.00638891
D1__Bacteria.D2__Cyanobacteria.D3__Oxyphotobacteria	2.952764608	P_WT	2.673081274	0.000126849	2.71399262	T_APP_PS1_5Xn	2.403099612	0.001807566
D1__Bacteria.D2__Cyanobacteria.D3__Oxyphotobacteria.D4__Chloroplast	2.20066104			0.000316771	2.246922377			0.001790432
D1__Bacteria.D2__Firmicutes.D3__Bacilli.D4__Bacillales.D5__Family_XII	2.353187944	P_WT	2.214832669	0.000185142	2.77171895	T_APP_PS1_5Xn	2.528483804	0.000689003
D1__Bacteria.D2__Firmicutes.D3__Bacilli.D4__Bacillales.D5__Family_XII.D6__Exiguobacterium	2.353187944	P_WT	2.161583902	0.000185142	2.77171895	T_APP_PS1_5Xn	2.528484165	0.000689003
**D1__Bacteria.D2__Firmicutes.D3__Bacilli.D4__Bacillales.D5__Planococcaceae**	2.313748786	**P_WT**	2.064274452	0.001135368	2.44603665	**T_WT_5Xn**	2.375239325	0.000918057
D1__Bacteria.D2__Firmicutes.D3__Erysipelotrichia.D4__Erysipelotrichales.D5__Erysipelotrichaceae.D6__Erysipelotrichaceae_UCG_004	1.375180593			0.003216155	1.705721339			0.006590879
D1__Bacteria.D2__Gemmatimonadetes.D3__Gemmatimonadetes.D4__Gemmatimonadales.D5__Gemmatimonadaceae.D6__Gemmatimonas	1.927151337			0.000120269	1.938745685			0.000957488
D1__Bacteria.D2__Proteobacteria.D3__Alphaproteobacteria.D4__Azospirillales	1.478979744			0.025095988	1.620220313			0.006400905
D1__Bacteria.D2__Proteobacteria.D3__Alphaproteobacteria.D4__Reyranellales	1.194029852			0.029229698	1.766332003			0.000121138
D1__Bacteria.D2__Proteobacteria.D3__Alphaproteobacteria.D4__Reyranellales.D5__Reyranellaceae	1.194029852			0.029229698	1.766332003			0.000121138
D1__Bacteria.D2__Proteobacteria.D3__Alphaproteobacteria.D4__Reyranellales.D5__Reyranellaceae.D6__Reyranella	1.194029852			0.029229698	1.728553869			0.000147636
D1__Bacteria.D2__Proteobacteria.D3__Alphaproteobacteria.D4__Rhizobiales	3.247386416	P_WT	2.9331368	0.001468789	3.025031582	T_APP_PS1_5Xn	2.707877068	0.044627909
D1__Bacteria.D2__Proteobacteria.D3__Alphaproteobacteria.D4__Rhizobiales.D5__Rhizobiales_Incertae_Sedis	2.731199512	P_WT	2.430500969	0.000233996	2.664640349	T_APP_PS1_5Xn	2.305760555	0.003279889
D1__Bacteria.D2__Proteobacteria.D3__Alphaproteobacteria.D4__Rhizobiales.D5__Rhizobiales_Incertae_Sedis.D6__Alsobacter	1.294328875			0.000140705	1.305834806			0.003118475
D1__Bacteria.D2__Proteobacteria.D3__Alphaproteobacteria.D4__Rhodobacterales	2.321436595	P_WT	2.054952358	9.43×10^{-5}	2.361470738	T_APP_PS1_5Xn	2.00764959	0.000681451
D1__Bacteria.D2__Proteobacteria.D3__Alphaproteobacteria.D4__Rhodobacterales.D5__Rhodobacteraceae	2.321436595	P_WT	2.054968596	9.43×10^{-5}	2.361470738	T_APP_PS1_5Xn	2.006898828	0.000681451
D1__Bacteria.D2__Proteobacteria.D3__Alphaproteobacteria.D4__Rhodobacterales.D5__Rhodobacteraceae.D6__Paracoccus	1.63144938			3.29×10^{-5}	1.395935058			0.001937176
D1__Bacteria.D2__Proteobacteria.D3__Alphaproteobacteria.D4__Rhodobacterales.D5__Rhodobacteraceae.D6__Tabrizicola	1.782877223			0.00287242	1.606057038			0.001864521
D1__Bacteria.D2__Proteobacteria.D3__Alphaproteobacteria.D4__Sphingomonadales	2.85003312	P_WT	2.561602956	0.00095516	2.793951845	T_APP_PS1_5Xn	2.4118532	0.008374721

D1__Bacteria.D2__Proteobacteria.D3__Alphaproteobacteria.D4__Sphingomonadales.D5__Sphingomonadaceae	2.85003312	P_WT	2.561469333	0.00095516	2.793951845	T_APP_PS1_5Xn	2.412875087	0.008374721
D1__Bacteria.D2__Proteobacteria.D3__Alphaproteobacteria.D4__Sphingomonadales.D5__Sphingomonadaceae.D6__Altererythrobacter	1.736491001			0.012719332	1.51189694			0.014544975
D1__Bacteria.D2__Proteobacteria.D3__Alphaproteobacteria.D4__Sphingomonadales.D5__Sphingomonadaceae.D6__Novosphingobium	2.24251111			0.001231625	2.218504705			0.042144929
**D1__Bacteria.D2__Proteobacteria.D3__Gammaproteobacteria**	4.340473504	P_APP_PS1	4.084995595	0.003259155	4.232125495	T_APP_PS1_5Xn	3.845280843	0.007554033
D1__Bacteria.D2__Proteobacteria.D3__Gammaproteobacteria.D4__Betaproteobacteriales.D5__Burkholderiaceae.D6__Limnohabitans	1.35980199			0.000586246	1.473801842			0.000664499
D1__Bacteria.D2__Proteobacteria.D3__Gammaproteobacteria.D4__Betaproteobacteriales.D5__Methylophilaceae	2.40454377	P_WT	2.161812377	0.00036888	2.216242729			0.013600085
D1__Bacteria.D2__Proteobacteria.D3__Gammaproteobacteria.D4__Betaproteobacteriales.D5__Nitrosomonadaceae	1.742222162			0.002900057	1.913012466			0.002639375
D1__Bacteria.D2__Proteobacteria.D3__Gammaproteobacteria.D4__Betaproteobacteriales.D5__Rhodocyclaceae	1.581762017			0.000182472	1.418388395			0.00256787
D1__Bacteria.D2__Proteobacteria.D3__Gammaproteobacteria.D4__Cellvibrionales	2.077537072			0.001236706	2.482804785	T_APP_PS1_5Xn	2.15068148	0.002225229
D1__Bacteria.D2__Proteobacteria.D3__Gammaproteobacteria.D4__Cellvibrionales.D5__Spongiibacteraceae	2.040228522			0.000182472	2.028428438			0.002626285
D1__Bacteria.D2__Proteobacteria.D3__Gammaproteobacteria.D4__Cellvibrionales.D5__Spongiibacteraceae.D6__BD1_7_clade	2.040228522			0.000182472	1.974510714			0.002622666
D1__Bacteria.D2__Proteobacteria.D3__Gammaproteobacteria.D4__Pseudomonadales.D5__Pseudomonadaceae	2.457716272	P_APP_PS1	2.261051525	0.015128343	2.100034301			0.011273603
D1__Bacteria.D2__Proteobacteria.D3__Gammaproteobacteria.D4__Pseudomonadales.D5__Pseudomonadaceae.D6__Pseudomonas	2.457716272	P_APP_PS1	2.262045156	0.015128343	2.100034301			0.011273603
D1__Bacteria.D2__Proteobacteria.D3__Gammaproteobacteria.D4__Steroidobacterales	1.741653363			0.001554545	1.58022256			0.010127667
D1__Bacteria.D2__Proteobacteria.D3__Gammaproteobacteria.D4__Steroidobacterales.D5__Steroidobacteraceae	1.741653363			0.001554545	1.529986923			0.021815973
D1__Bacteria.D2__Proteobacteria.D3__Gammaproteobacteria.D4__Steroidobacterales.D5__Steroidobacteraceae.D6__uncultured	1.712669838			0.001056715	1.514112928			0.004343963
D1__Bacteria.D2__Verrucomicrobia.D3__Verrucomicrobiae.D4__Chthoniobacterales	2.20691232			2.05×10^{-5}	2.32743439	T_APP_PS1_5Xn	2.106334753	0.000812008
D1__Bacteria.D2__Verrucomicrobia.D3__Verrucomicrobiae.D4__Chthoniobacterales.D5__Chthoniobacteraceae	2.128963402			$2.05E \times 10^{-5}$	2.279194171	T_APP_PS1_5Xn	2.056872985	0.000746171
D1__Bacteria.D2__Verrucomicrobia.D3__Verrucomicrobiae.D4__Opitutales	1.95125095			0.000870253	1.771936832			0.003755453
D1__Bacteria.D2__Verrucomicrobia.D3__Verrucomicrobiae.D4__Verrucomicrobiales.D5__Rubritaleaceae	1.778986936			0.004130602	1.743081835			0.002036742
D1__Bacteria.D2__Verrucomicrobia.D3__Verrucomicrobiae.D4__Verrucomicrobiales.D5__Rubritaleaceae.D6__Luteolibacter	1.778986936			0.004130602	1.633520928			0.002461392

These differential species were identified with LDA Scores > 2.0 are based on Kruskal-Wallis test and Wilcoxon test. Group indicates a high abundance of differential species and plays an important role in this group. D1, D2, D3, D4, D5 and D6 represent Domain, Phylum, Class, Order, Family and Genus, respectively. Black bold words indicate selected representative differential species. LDA, Line Discriminant Analysis. LEfSe, LDA Effect Size.

Table S2 Abundance comparison of differential bacteria on Family level in the prevention and therapeutic experiments.

Bacteria Name	P_WT	P_WT_5Xn	P_APP_PS1	P_APP_PS1_5Xn	*p* value	Regulation	T_WT	T_WT_5Xn	T_APP_PS1	T_APP_PS1_5Xn	*p* value	Regulation
0319-6G20	0.02149	0.00378	0.00312	0.00000	0.00023	down	0.00000	0.00202	0.00322	0.00953	0.00195	up
67-14	0.00699	0.00358	0.00055	0.00000	0.00019	down	0.00061	0.00036	0.00274	0.01454	0.00881	irregular
Acetobacteraceae	0.01908	0.00213	0.00037	0.00000	0.00005	down	0.00012	0.00155	0.00972	0.01164	0.00560	up
Akkermansiaceae	9.00669	6.14889	4.98760	0.76721	0.02367	down	0.10768	0.60356	0.02444	5.47819	0.03048	up
AKYH767	0.01397	0.00318	0.00018	0.00000	0.00007	down	0.00000	0.00071	0.00418	0.01224	0.00066	up
**Alteromonadaceae**	**0.02105**	**0.00080**	**0.00057**	**0.00000**	0.00030	**down**	**0.00014**	**0.00226**	**0.00585**	**0.00457**	0.01155	**down**
Balneolaceae	0.02669	0.00398	0.00165	0.00000	0.00006	down	0.00014	0.00166	0.01139	0.02114	0.00392	up
Barnesiellaceae	0.00287	0.00159	0.00018	0.00000	0.02058	down	0.00000	0.00036	0.00065	0.01444	0.00752	up
Bdellovibrionaceae	0.00752	0.00000	0.00000	0.00000	0.00000	down	0.00000	0.00024	0.00302	0.00427	0.00476	up
Burkholderiaceae	0.18532	0.11719	0.19727	0.06831	0.03427	down	0.09583	0.15678	0.20244	1.24573	0.03496	up
Caldilineaceae	0.01304	0.00298	0.00037	0.00000	0.00007	down	0.00000	0.00285	0.00388	0.00574	0.00977	up
Chitinophagaceae	0.04131	0.00895	0.00514	0.00000	0.00019	down	0.00178	0.00380	0.01383	0.03470	0.00914	up
Chthoniobacteraceae	0.01325	0.00199	0.00000	0.00000	0.00002	down	0.00000	0.00095	0.00500	0.01873	0.00075	up
Clade_III	0.04865	0.01074	0.00532	0.00000	0.00023	down	0.00014	0.00190	0.01786	0.03825	0.00208	up
Crocinitomicaceae	0.01533	0.00357	0.00092	0.00000	0.00021	down	0.00000	0.00107	0.00433	0.00988	0.00354	up
Cryomorphaceae	0.05034	0.00676	0.00642	0.00000	0.00015	down	0.00000	0.00197	0.01528	0.03032	0.00065	up
Cyanobiaceae	0.04988	0.01412	0.00183	0.00000	0.00012	down	0.00000	0.00430	0.01892	0.03117	0.00336	up
Cyclobacteriaceae	0.02719	0.00497	0.00440	0.00000	0.00015	down	0.00056	0.00257	0.00837	0.02748	0.00114	up
env.OPS_17	0.00675	0.00080	0.00000	0.00000	0.00090	down	0.00000	0.00012	0.00147	0.00544	0.00072	up
Family_XII	0.02218	0.02028	0.00092	0.00000	0.00019	down	0.00000	0.00321	0.00958	0.05877	0.00018	up
Fusobacteriaceae	0.02127	0.00020	0.00244	0.00079	0.00024	down	0.00013	0.01344	0.00784	0.01098	0.00363	up
Gemmatimonadaceae	0.05668	0.01033	0.00330	0.00027	0.00022	down	0.00013	0.00392	0.01827	0.04586	0.00055	up
Ilumatobacteraceae	0.09331	0.01370	0.00807	0.00000	0.00014	down	0.00071	0.00815	0.03236	0.07256	0.00146	up

JG30-KF-CM45	0.00341	0.00040	0.00092	0.00000	0.00105	down	0.00000	0.00024	0.00039	0.00146	0.00639	up
Legionellaceae	0.00389	0.00020	0.00055	0.00000	0.00030	down	0.00000	0.00024	0.00329	0.00378	0.01161	up
Metagenome	0.08792	0.02530	0.01701	0.00167	0.00274	down	0.00026	0.00857	0.02848	0.08624	0.00329	up
Methylophilaceae	0.02508	0.00507	0.00220	0.00000	0.00037	down	0.00333	0.00449	0.00722	0.01624	0.01360	up
Microbacteriaceae	0.05268	0.04295	0.01256	0.00000	0.00077	down	0.00132	0.00488	0.01968	0.04361	0.01883	up
Microtrichaceae	0.00339	0.00159	0.00000	0.00000	0.00805	down	0.00000	0.00095	0.00208	0.00674	0.00687	up
Mycobacteriaceae	0.01211	0.00398	0.00202	0.00000	0.00228	down	0.00076	0.00143	0.00546	0.00873	0.00890	up
**Nitriliruptoraceae**	**0.00416**	**0.00264**	**0.00078**	**0.00024**	0.04522	**down**	0.00000	0.00000	0.00243	0.00235	0.00029	**down**
Nitrosomonadaceae	0.00543	0.00060	0.00018	0.00000	0.00290	down	0.00000	0.00086	0.00263	0.00856	0.00240	up
**Nodosilineaceae**	**0.01087**	**0.00099**	**0.00073**	**0.00000**	0.00046	**down**	0.00000	0.00000	0.00554	0.00133	0.02483	**down**
NS11-12_marine_group	0.05873	0.00794	0.00110	0.00000	0.00009	down	0.00027	0.00440	0.02222	0.05408	0.00367	up
NS9_marine_group	0.02708	0.01670	0.00128	0.00000	0.00015	down	0.00000	0.00464	0.00592	0.00676	0.00377	up
Oligoflexaceae	0.05664	0.01014	0.00275	0.00000	0.00008	down	0.00000	0.00820	0.01842	0.01988	0.00324	up
Opitutaceae	0.00884	0.00378	0.00128	0.00000	0.00036	down	0.00000	0.00012	0.00261	0.00531	0.01121	up
Pedosphaeraceae	0.00315	0.00080	0.00018	0.00000	0.00345	down	0.00000	0.00107	0.00212	0.00411	0.00776	up
Phycisphaeraceae	0.00678	0.00099	0.00018	0.00000	0.00005	down	0.00000	0.00000	0.00327	0.00425	0.00471	up
Planococcaceae	0.02022	0.00737	0.00055	0.00000	0.00114	down	0.00048	0.02785	0.00559	0.02047	0.00086	up
Pseudohongiellaceae	0.02399	0.00358	0.00202	0.00000	0.00008	down	0.00000	0.00074	0.01050	0.01348	0.00295	up
Pseudomonadaceae	0.02390	0.00387	0.02818	0.00071	0.01513	down	0.00055	0.00159	0.00481	0.01237	0.01127	up
Reyranellaceae	0.00155	0.00040	0.00000	0.00000	0.02923	down	0.00000	0.00000	0.00050	0.00575	0.00012	up
Rhizobiales_Incertae_Sedis	0.05307	0.00696	0.00752	0.00000	0.00023	down	0.00111	0.00668	0.01874	0.04550	0.00328	up
Rhodobacteraceae	0.05193	0.00894	0.00385	0.00000	0.00016	down	0.00027	0.00722	0.01866	0.03715	0.00089	up
Rhodocyclaceae	0.00456	0.00060	0.00000	0.00000	0.00015	down	0.00000	0.00083	0.00078	0.00259	0.00457	up
**Rhodospirillaceae**	**0.00552**	**0.00139**	**0.00073**	**0.00000**	0.00029	**down**	**0.00000**	**0.00000**	**0.00186**	**0.00089**	0.02520	**down**
Rickettsiaceae	0.01146	0.00159	0.00037	0.00000	0.00006	down	0.00000	0.00107	0.00437	0.00573	0.00325	up
Roseiflexaceae	0.01303	0.00179	0.00000	0.00000	0.00003	down	0.00012	0.00036	0.00302	0.00663	0.00135	up

Rubritaleaceae	0.00595	0.00219	0.00018	0.00000	0.00413	down	0.00000	0.00028	0.00144	0.00547	0.00185	up
Saprospiraceae	0.08554	0.01590	0.00367	0.00000	0.00011	down	0.00000	0.00813	0.02652	0.06638	0.00073	up
Shewanellaceae	0.00347	0.00139	0.00018	0.00000	0.00056	down	0.00000	0.00048	0.00105	0.00150	0.04958	up
Sphingomonadaceae	0.07069	0.00742	0.00870	0.00027	0.00096	down	0.00702	0.00562	0.01546	0.06139	0.00852	irregular
Spirosomaceae	0.01006	0.00358	0.00202	0.00000	0.00859	down	0.00041	0.00083	0.00131	0.00666	0.01353	up
Spongiibacteraceae	0.01079	0.00278	0.00000	0.00000	0.00018	down	0.00000	0.00475	0.00446	0.01154	0.00288	up
Sporichthyaceae	0.15490	0.01511	0.02109	0.00000	0.00008	down	0.00013	0.00856	0.05372	0.08361	0.00457	up
Steroidobacteraceae	0.00545	0.00080	0.00165	0.00000	0.00155	down	0.00119	0.00107	0.00078	0.00335	0.02182	up
Terrimicrobiaceae	0.00259	0.00040	0.00000	0.00000	0.00568	down	0.00000	0.00000	0.00041	0.00164	0.01061	up
**Uncultured**	**0.03450**	**0.03208**	**0.01274**	**0.02335**	0.04241	**up**	**0.00756**	**0.03065**	**0.01028**	**0.08188**	0.00038	**up**
uncultured_Acidimicrobidae_bacterium	0.00078	0.00000	0.00000	0.00000	0.02659	down	0.00000	0.00000	0.00000	0.00135	0.02455	up
**uncultured_bacterium**	0.17711	0.07874	**0.04677**	**0.09666**	0.02678	irregular	0.03941	0.15819	**0.09233**	**0.27353**	0.00230	up
uncultured_Bacteroidetes_bacterium	0.00672	0.00099	0.00018	0.00000	0.00045	down	0.00000	0.00107	0.00202	0.02240	0.00021	up
Unknown_Family	0.03200	0.00338	0.00486	0.00000	0.00010	down	0.00131	0.00226	0.01123	0.01944	0.00163	up
Verrucomicrobiaceae	0.00608	0.00338	0.00018	0.00000	0.00084	down	0.00012	0.00107	0.00145	0.00534	0.04123	up
Vibrionaceae	0.01899	0.00537	0.00257	0.00000	0.00029	down	0.00000	0.00112	0.00428	0.00562	0.03865	up
Virgulinella_fragilis	0.00896	0.00159	0.00018	0.00000	0.00287	down	0.00000	0.00276	0.00209	0.00612	0.00593	up

Abundance comparison of differential bacteria was done via Kruskal-Wallis test. Black boldface words indicate the bacteria with the same regulation tendency after Xn treatment.

Table S3 Abundance comparison of differential bacteria on Genus level in the prevention and therapeutic experiments.

Bacteria Name	P_WT	P_WT_5Xn	P_APP_PS1	P_APP_PS1_5Xn	*p* value	Regulation	T_WT	T_WT_5Xn	T_APP_PS1	T_APP_PS1_5Xn	*p* value	Regulation
Acidibacter	0.02894	0.00338	0.00468	0.00000	0.00015	down	0.00131	0.00226	0.01071	0.01701	0.00588	up
Akkermansia	9.00669	6.14889	4.98760	0.76721	0.02367	down	0.10768	0.60356	0.02444	5.47819	0.03048	up
Algoriphagus	0.01533	0.00219	0.00275	0.00000	0.00019	down	0.00000	0.00155	0.00352	0.01779	0.00105	up
Alsobacter	0.00194	0.00000	0.00000	0.00000	0.00014	down	0.00000	0.00024	0.00118	0.00199	0.00312	up
Altererythrobacter	0.00540	0.00060	0.00018	0.00000	0.01272	down	0.00012	0.00097	0.00182	0.00322	0.01454	up
Arenimonas	0.00151	0.00000	0.00018	0.00000	0.02139	down	0.00000	0.00000	0.00091	0.00959	0.03728	up
bacterium_enrichment_culture _clone_B126(2011)	0.01929	0.00338	0.00312	0.00000	0.00023	down	0.00000	0.00119	0.00241	0.00885	0.00172	up
BD1-7_clade	0.01079	0.00278	0.00000	0.00000	0.00018	down	0.00000	0.00276	0.00446	0.00933	0.00262	up
Bdellovibrio	0.00734	0.00000	0.00000	0.00000	0.00000	down	0.00000	0.00024	0.00224	0.00335	0.00525	up
Candidatus_Aquiluna	0.03699	0.00358	0.00862	0.00000	0.00015	down	0.00028	0.00321	0.01420	0.03547	0.00254	up
Candidatus_Chloroploca	0.00227	0.00020	0.00018	0.00000	0.01071	down	0.00014	0.00000	0.00040	0.00157	0.04048	irregular
Candidatus_Megaira	0.01146	0.00159	0.00037	0.00000	0.00006	down	0.00000	0.00107	0.00410	0.00545	0.00309	up
Candidatus_Methylopumilus	0.00904	0.00239	0.00147	0.00000	0.00023	down	0.00000	0.00155	0.00261	0.00638	0.00852	up
Cephaloticoccus	0.00255	0.00020	0.00037	0.00000	0.00423	down	0.00000	0.00000	0.00104	0.00161	0.01603	up
Cetobacterium	0.02127	0.00020	0.00169	0.00024	0.00009	down	0.00013	0.01333	0.00733	0.01053	0.01657	up
Chryseomicrobium	0.00377	0.00179	0.00018	0.00000	0.00068	down	0.00000	0.00037	0.00104	0.00806	0.01964	up
CK06-06-Mud-MAS4B-21	0.01147	0.00199	0.00037	0.00000	0.00006	down	0.00014	0.00012	0.00441	0.00996	0.00227	up
CL500-29_marine_group	0.07981	0.01054	0.00660	0.00000	0.00010	down	0.00059	0.00654	0.02831	0.06328	0.00322	up
CL500-3	0.00678	0.00099	0.00018	0.00000	0.00005	down	0.00000	0.00000	0.00327	0.00425	0.00471	up
Comamonas	0.00212	0.00019	0.00018	0.00000	0.04791	down	0.00014	0.00052	0.00200	0.00459	0.02347	up
Cyanobium_PCC-6307	0.04881	0.01392	0.00183	0.00000	0.00015	down	0.00000	0.00416	0.01879	0.03093	0.00190	up
Dinghuibacter	0.01716	0.00199	0.00147	0.00000	0.00006	down	0.00000	0.00095	0.00613	0.01532	0.00108	up

Erysipelotrichaceae_UCG-004	0.00236	0.00000	0.00018	0.00000	0.00322	down	0.00000	0.00000	0.00132	0.00500	0.00659	up
Eubacterium_brachy_group	0.02660	0.01759	0.05078	0.09433	0.01966	irregular	0.02902	0.02126	0.05266	0.05396	0.04170	irregular
Exiguobacterium	0.02218	0.02028	0.00092	0.00000	0.00019	down	0.00000	0.00321	0.00958	0.05835	0.00069	up
Fluviicola	0.01314	0.00357	0.00073	0.00000	0.00026	down	0.00000	0.00024	0.00394	0.00936	0.00927	up
Gemmatimonas	0.00836	0.00139	0.00073	0.00000	0.00012	down	0.00000	0.00024	0.00267	0.00854	0.00096	up
Gordonibacter	0.00143	0.01894	0.00884	0.00663	0.01815	irregular	0.00890	0.02177	0.03741	0.03555	0.04867	irregular
Haliscomenobacter	0.01658	0.00258	0.00000	0.00000	0.00110	down	0.00000	0.00250	0.00539	0.00695	0.02294	up
HdN1	0.00213	0.00000	0.00000	0.00000	0.00533	down	0.00000	0.00000	0.00106	0.00129	0.00438	up
hgcI_clade	0.14736	0.01292	0.02054	0.00000	0.00008	down	0.00013	0.00808	0.05109	0.07348	0.00457	up
Jeotgalibacillus	0.00499	0.00060	0.00000	0.00000	0.00090	down	0.00000	0.00095	0.00264	0.00226	0.02377	irregular
**Lachnospiraceae_UCG-001**	**0.18847**	**0.02420**	**0.16229**	**0.11142**	0.04728	**down**	**0.27274**	**0.03543**	**0.15229**	**0.03189**	0.00343	**down**
Lacihabitans	0.00197	0.00000	0.00000	0.00000	0.02659	down	0.00000	0.00024	0.00000	0.00213	0.00525	up
Lautropia	0.00455	0.00080	0.00037	0.00000	0.00423	down	0.00000	0.00000	0.00065	0.00264	0.02483	up
LD29	0.01305	0.00179	0.00000	0.00000	0.00002	down	0.00000	0.00095	0.00500	0.01112	0.00117	up
Legionella	0.00351	0.00020	0.00055	0.00000	0.00037	down	0.00000	0.00024	0.00316	0.00366	0.01207	up
Limnobacter	0.01872	0.00298	0.00073	0.00000	0.00006	down	0.00000	0.00178	0.00525	0.00974	0.00190	up
Limnohabitans	0.00227	0.00000	0.00018	0.00000	0.00059	down	0.00000	0.00000	0.00080	0.00294	0.00066	up
Luteolibacter	0.00595	0.00219	0.00018	0.00000	0.00413	down	0.00000	0.00014	0.00144	0.00425	0.00224	up
Metagenome	0.11946	0.03305	0.01866	0.00167	0.00280	down	0.00190	0.01309	0.03513	0.11199	0.01682	up
MWH-UniP1_aquatic_group	0.04718	0.00338	0.00312	0.00000	0.00004	down	0.00000	0.00357	0.02947	0.03634	0.00226	up
Mycobacterium	0.01211	0.00398	0.00202	0.00000	0.00228	down	0.00076	0.00143	0.00546	0.00873	0.00890	up
**Nodosilinea_PCC-7104**	0.01030	0.00080	**0.00073**	**0.00000**	0.00057	**down**	0.00000	0.00000	**0.00489**	**0.00133**	0.02483	**down**
Noviherbaspirillum	0.00248	0.00040	0.00000	0.00000	0.00679	down	0.00000	0.00024	0.00093	0.00129	0.01781	up
Novosphingobium	0.01723	0.00326	0.00203	0.00027	0.00123	down	0.00462	0.00144	0.00536	0.01634	0.04214	irregular
NS10_marine_group	0.00322	0.00199	0.00000	0.00000	0.00805	down	0.00000	0.00012	0.00053	0.00686	0.00109	up
oc32	0.00097	0.00000	0.00000	0.00000	0.02659	down	0.00000	0.00024	0.00052	0.00122	0.04834	up

OM43_clade	0.00437	0.00020	0.00055	0.00000	0.00046	down	0.00000	0.00048	0.00236	0.00295	0.00584	up
Paracoccus	0.00421	0.00119	0.00000	0.00000	0.00003	down	0.00000	0.00119	0.00094	0.00246	0.00194	up
Peredibacter	0.00562	0.00000	0.00000	0.00000	0.00014	down	0.00000	0.00000	0.00108	0.00112	0.04144	up
Perlucidibaca	0.02897	0.00099	0.00110	0.00000	0.00002	down	0.00000	0.00262	0.01479	0.01629	0.00879	up
Phaeodactylibacter	0.00444	0.00020	0.00000	0.00000	0.01810	down	0.00000	0.00024	0.00197	0.00354	0.02715	up
Polynucleobacter	0.00240	0.00040	0.00000	0.00000	0.00018	down	0.00000	0.00024	0.00226	0.00434	0.00410	up
**Prevotella_9**	0.00000	0.00075	**0.00000**	**0.00051**	0.04506	up	0.00215	0.00000	**0.00047**	**0.28009**	0.02007	irregular
Pseudohongiella	0.02399	0.00358	0.00202	0.00000	0.00008	down	0.00000	0.00074	0.01050	0.01348	0.00295	up
Pseudomonas	0.02390	0.00387	0.02818	0.00071	0.01513	down	0.00055	0.00159	0.00481	0.01237	0.01127	up
Reyranella	0.00155	0.00040	0.00000	0.00000	0.02923	down	0.00000	0.00000	0.00050	0.00526	0.00015	up
Rheinheimera	0.01285	0.00040	0.00000	0.00000	0.00007	down	0.00014	0.00119	0.00453	0.00308	0.02787	irregular
**Rikenella**	0.06189	0.08648	**0.27206**	**0.03600**	0.01836	irregular	0.05988	0.04465	**0.22151**	**0.14316**	0.03122	down
Roseomonas	0.01334	0.00099	0.00018	0.00000	0.00002	down	0.00012	0.00036	0.00761	0.01005	0.00300	up
Rubellimicrobium	0.00292	0.00020	0.00018	0.00000	0.00239	down	0.00000	0.00012	0.00066	0.00487	0.01393	up
Rubrivivax	0.02286	0.00418	0.00073	0.00000	0.00006	down	0.00095	0.00273	0.00639	0.00994	0.01992	up
Ruminiclostridium	0.10911	0.03148	0.15168	0.40615	0.00319	irregular	0.23312	0.15394	0.28351	0.06192	0.00315	down
Sandarakinorhabdus	0.00476	0.00000	0.00018	0.00000	0.00047	down	0.00000	0.00071	0.00237	0.00235	0.02001	up
Sediminibacterium	0.00560	0.00099	0.00000	0.00000	0.00015	down	0.00000	0.00048	0.00196	0.01008	0.00366	up
Shewanella	0.00347	0.00139	0.00018	0.00000	0.00056	down	0.00000	0.00048	0.00105	0.00150	0.04958	up
Sva0996_marine_group	0.00162	0.00139	0.00000	0.00000	0.03380	down	0.00000	0.00081	0.00117	0.00485	0.00776	up
Tabrizicola	0.00599	0.00119	0.00018	0.00000	0.00287	down	0.00000	0.00155	0.00119	0.00399	0.00186	up
Terrimicrobium	0.00259	0.00040	0.00000	0.00000	0.00568	down	0.00000	0.00000	0.00041	0.00164	0.01061	up
Turicibacter	0.00078	0.09521	0.00303	0.00464	0.02516	up	0.15240	0.12339	0.00561	0.00096	0.00838	down
uncultured_Acidimicrobidae_bacterium	0.00078	0.00000	0.00000	0.00000	0.02659	down	0.00000	0.00000	0.00000	0.00135	0.02455	up
uncultured_actinobacterium	0.00252	0.00128	0.00000	0.00000	0.00995	down	0.00000	0.00000	0.00028	0.00784	0.00589	up

uncultured_Bacteroidetes_bacterium	0.01061	0.00119	0.00018	0.00000	0.00045	down	0.00000	0.00166	0.00242	0.02656	0.00022	up
uncultured_Flexibacteraceae_bacterium	0.00763	0.00119	0.00000	0.00000	0.00110	down	0.00000	0.00012	0.00380	0.00760	0.01335	up
uncultured_gamma_proteobacterium	0.00298	0.00139	0.00165	0.00000	0.03845	down	0.00000	0.00036	0.00053	0.00119	0.02996	up
uncultured_marine_bacterium	0.00911	0.00060	0.00037	0.00000	0.00037	down	0.00000	0.00036	0.00568	0.00549	0.00056	irregular
uncultured_Sphingobacterium_sp.	0.02106	0.00139	0.00018	0.00000	0.00002	down	0.00000	0.00190	0.00608	0.01660	0.00158	up
Vibrio	0.01899	0.00537	0.00257	0.00000	0.00029	down	0.00000	0.00112	0.00402	0.00562	0.03865	up
Virgulinella_fragilis	0.00896	0.00159	0.00018	0.00000	0.00287	down	0.00000	0.00276	0.00209	0.00612	0.00593	up

Abundance comparison of differential bacteria was done via the Kruskal-Wallis test. Black boldface words indicate the bacteria with the same regulation tendency after Xn treatment.
